# LSS-RM: Using Multi-Mounted Devices to Construct a Lightweight Site-Survey Radio Map for WiFi Positioning

**DOI:** 10.3390/mi9090458

**Published:** 2018-09-12

**Authors:** Wei Yang, Chundi Xiu, Jiarui Ye, Zhixing Lin, Haisong Wei, Dayu Yan, Dongkai Yang

**Affiliations:** School of Electronic and Information Engineering, Beihang University, Beijing 100083, China; yangwei89@buaa.edu.cn (W.Y.); yejiarui@buaa.edu.cn (J.Y.); linzhixing@buaa.edu.cn (Z.L.); sy1702122@buaa.edu.cn (H.W.); dyaxb@buaa.edu.cn (D.Y.); edkyang@buaa.edu.cn (D.Y.)

**Keywords:** indoor positioning, WiFi-RSSI radio map, MEMS-IMU accelerometer, zero-velocity update, step detection, stride length estimation

## Abstract

A WiFi-received signal strength index (RSSI) fingerprinting-based indoor positioning system (WiFi-RSSI IPS) is widely studied due to advantages of low cost and high accuracy, especially in a complex indoor environment where performance of the ranging method is limited. The key drawback that limits the large-scale deployment of WiFi-RSSI IPS is time-consuming offline site surveys. To solve this problem, we developed a method using multi-mounted devices to construct a lightweight site-survey radio map (LSS-RM) for WiFi positioning. A smartphone was mounted on the foot (Phone-F) and another on the waist (Phone-W) to scan WiFi-RSSI and simultaneously sample microelectromechanical system inertial measurement-unit (MEMS-IMU) readings, including triaxial accelerometer, gyroscope, and magnetometer measurements. The offline site-survey phase in LSS-RM is a client–server model of a data collection and preprocessing process, and a post calibration process. Reference-point (RP) coordinates were estimated using the pedestrian dead-reckoning algorithm. The heading was calculated with a corner detected by Phone-W and the preassigned site-survey trajectory. Step number and stride length were estimated using Phone-F based on the stance-phase detection algorithm. Finally, the WiFi-RSSI radio map was constructed with the RP coordinates and timestamps of each stance phase. Experimental results show that our LSS-RM method can reduce the time consumption of constructing a WiFi-RSSI radio map from 54 min to 7.6 min compared with the manual site-survey method. The average positioning error was below 2.5 m with three rounds along the preassigned site-survey trajectory. LSS-RM aims to reduce offline site-survey time consumption, which would cut down on manpower. It can be used in the large-scale implementation of WiFi-RSSI IPS, such as shopping malls, hospitals, and parking lots.

## 1. Introduction

The positioning method is a basic component of location-based services (LBSs) such as navigating a customer to the nearest restaurant in a shopping mall, finding your car in an underground parking lot, guiding tourists in a museum, or aiding during a fire emergency [1]. As to positioning outdoors, global navigation satellite systems (GNSSs) can provide global services and users can get an accurate position, velocity, and time (PVT) in open air [2,3]. However, in an indoor environment and urban canyons, GNSS signal availability is limited, and indoor-positioning systems (IPSs) need to be studied [4].

IPSs can be classified roughly into two kinds: the infrastructure-free system and the infrastructure-based system [5]. A typical infrastructure-free system is the inertial navigation system (INS) [6]. Pedestrian dead-reckoning (PDR)-based INS (PDR-INS) utilizes the pedestrian kinetic model to do step detection, stride-length estimation, and heading estimation [7,8,9,10]. Considering the drift of gyroscope readings and the fluctuation of indoor magnetic fields, an accurate heading estimation is difficult, and map information, including walls, corridors, and rooms, can be fused using a particle filter to get more accurate heading estimation [11]. The positioning accuracy of PDR-INS is easily influenced by the carry mode of devices, and the stride-length model needs parameters like height, leg length, or walking frequency, which should be tuned according to different users [12]. In some other pedestrian inertial navigation systems, especially for fire-emergency applications, microelectromechanical system inertial-measurement units (MEMS-IMU) are mounted on the foot. With the triaxial accelerometer and gyroscope readings, a zero-velocity update (ZUPT) algorithm is developed to measure velocity errors in the stance phase of a gait cycle [13,14]. ZUPT estimates pseudo measurements into the extended Kalman filter (EKF) navigation-error corrector, which allows the EKF to correct velocity errors during each gait cycle, breaking the cubic-in-time error growth and replacing it with an error accumulation that is linear with the number of steps [15,16,17]. This kind of foot-mounted inertial navigation system is called IEZ-INS and IEZ is short for the first letters of INS, EKF, and ZUPT. Foot-mounted MEMS-IMU can be used to get more accurate stride-length estimation [18,19,20]. There is no clear boundary between PDR-INS and IEZ-INS. The principle of method choice depends on the accuracy of MEMS-IMU and the specific application.

Unlike infrastructure-free IPSs, infrastructure-based ones need preinstalled transmitters such as WiFi [9,21,22], Bluetooth [23,24], near-field communication [25], RFID [26], ultrawide band (UWB) [27,28], LED [29,30], or ultrasound [31]. These methods can provide sufficient positioning accuracy for LBSs in different applications. Widely deployed private- or public-access points (APs) in large-scale buildings can provide free and dense signals for WiFi-based IPS. Furthermore, smartphones are embedded with WiFi chips that can easily obtain a received signal-strength index (RSSI). The methods used in WiFi-based IPS can be classified by time of arrival (TOA) [32], time difference of arrival (TDOA) [33], angle of arrival (AOA) [34], and RSSI fingerprinting [35], CSI-fingerprinting [36], and round-trip time (RTT) [37]. Each of these methods has shortcomings and limitations. TOA, TDOA, and AOA are easily influenced by indoor environments. Channel state information (CSI) fingerprinting needs an Intel 5300 wireless local area networks (WLAN) card that is not available for smartphones. Similar with CSI fingerprinting, the AP of the RTT method must support IEEE 802.11 mc, which is brand-new and not in the commercial market yet. Therefore, considering the complex multipath effect and the available hardware, RSSI fingerprinting is widely researched.

The WiFi-fingerprinting method consists of two phases, the offline site-survey phase and the online positioning phase [38]. In the offline phase, the WiFi-RSSI of selected APs is collected from each reference point (RP), and a radio map is built up. In the online phase, the collected WiFi-RSSI samples are compared with the radio map using matching algorithms such as k-nearest neighbor (KNN) to get the position estimation [39]. One of the most important reasons that limit the large-scale implementation of WiFi-based IPS is that site surveys are very time-consuming and labor-intensive [40]. For example, if we wanted to deploy the WiFi-IPS in a 10 m × 10 m room with a one-meter interval of each RP, and the WiFi-RSSI sampling time was 2 min of each RP, it would take 200 min to build the WiFi-RSSI radio map of this room in total. Time consumption would rise rapidly with the area of the place deploying WiFi-IPS [41].

To make WiFi-based IPS more practical, many researchers have focused on how to build the radio map in an energy-efficient way. The most common format of a WiFi-RSSI radio map is {RP coordinates, WiFi-RSSI vectors}. The offline WiFi-RSSI radio-training phase is very time-consuming because volunteers must stand still for a while to collect WiFi-RSSI from every RP [42]. Therefore, methods aiming to reduce the manpower of building a WiFi-RSSI radio map either research the model of WiFi-RSSI or add extra devices and sensors to help estimate RP coordinates [38,40]. According to Reference [43], methods that try to replace the construction of the radio map by using indoor radio-propagation models cannot capture all the details of the indoor structure and dynamics. These methods either achieve a very unsatisfactory performance or rectify model inaccuracies through extensive calibration and exhaustive postprocessing to obtain a map that can achieve more acceptable localization accuracy. In Reference [44], a WiFi-enabled laptop and an attached GPS device were used to scan the WiFi-RSSI in the metropolitan-scale area. RP coordinates were provided by the GPS device recording the latitude–longitude coordinates of the war driver when the WiFi-RSSI scan was performed. However, this method is limited indoors, and large GPS positioning is not accurate enough for calculating RP coordinates. In Reference [45], nanoscale unmanned aerial vehicles (UAVs) were used to automate the WiFi-RSSI collection process. RP coordinates were calculated through a UWB-based localization subsystem. This method can build a 3D WiFi-RSSI radio map with low manpower. With the high accuracy of UWB-based IPS, the performance of an automatically built WiFi-RSSI radio map is similar with the manually built one. However, it needs extra equipment, like UAVs and UWB anchors that increase the cost.

With the rapid development of the electronic industry, a state-of-the-art smartphone is a good platform embedded with multiple sensors like accelerometers, gyroscopes, and magnetometers to measure pedestrian movement, and WiFi chips to scan WiFi-RSSI. In Reference [46], a method called crowdsourcing and multisource fusion-based fingerprint sensing (CMFS) was presented. Based on the floor plan, RSSI is collected uniformly by WiFi scanner at fixed time intervals. Simultaneously, stride length, step number, and heading direction of volunteers were estimated using PDR-INS method. The drawback of this method is the drawback of PDR-INS. Stride-length parameters like pedestrian height and step frequency must be tuned according to different volunteers. In addition, heading direction is calculated using magnetic-field strength that may have non-negligible bias indoors. In Reference [47], a WiFi-RSSI radio map was built using an inertial navigation solution from a Trusted Portable Navigator (T-PN) with handheld smartphones. The method of calculating RP coordinates was T-PN, which improves the accuracy of RP coordinates with absolute measurements like A-GPS, magnetometer, or barometer as filter updates. However, the accuracy of T-PN decreases indoors. In Reference [48], a zero-effort crowdsourcing method (Zee) was developed. Zee used inertial sensors of smartphones and detailed map information to count steps and estimate heading offset. An augmented particle filter was utilized to estimate stride length with map information. Then, WiFi-RSSI was recorded to the radio map with RP coordinates through the same timestamps. However, Zee needs detailed map information that is not always available.

In this paper, we proposed a method called LSS-RM, short for Lightweight Site-Survey Radio Map, to construct the WiFi-RSSI radio map. This method, which can scan WiFi-RSSI, can significantly reduce the time consumption of offline WiFi-RSSI radio-map construction. Similar with other WiFi fingerprinting-based systems, our method also consists of an offline site-survey phase and online-positioning phase. The offline phase of LSS-RM is divided into the data collection and preprocessing process, and the post calibration process. The site survey is a client–server model in which the WiFi-based IPS service provider hires some volunteers to participate in site-survey work. To attract more volunteers to a boring site survey, the incentive mechanisms developed in Reference [49] can be applied.

The remainder of this paper is organized as follows: Section 2 is the overview of our WiFi-RSSI LSS-RM. In Section 3, the data collection and preprocessing processes are described. In Section 4, the post calibration process is described. In Section 5, experiments were performed, and the performance of the WiFi-RSSI radio map built with our method was evaluated. Finally, Section 6 concludes this work and offers future research suggestions.

## 2. Overview of LSS-RM

The WiFi-RSSI radio map consists of RP coordinates and a WiFi-RSSI vector. In the traditional manual site survey, a floor plan with detailed RP coordinates is provided to trained persons who are familiar with the site-survey process, usually from the service-provider group. Then, site-survey participants stand on each RP to scan WiFi-RSSI using smartphones. The most time-consuming part of the site survey is the manual WiFi-RSSI scan process. In this paper, site-survey participants did not need to stand still at each RP, instead walking along the preassigned site-survey trajectory, and the WiFi-RSSI radio map was automatically constructed.

**Figure 1:**
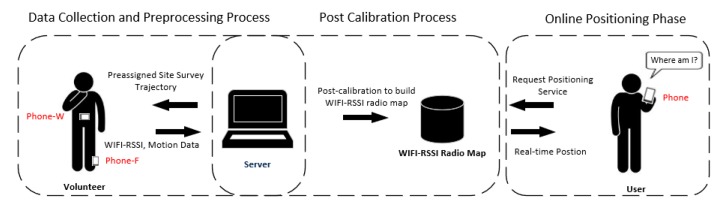
Structure of our WiFi-received signal strength index (WiFi-RSSI) radio map construction method with a lightweight site survey (LSS-RM). It consists of an offline site-survey phase and online-positioning phase. The offline site survey in LSS-RM has two processes: data collection and preprocessing process, and the post calibration processes. A WiFi-RSSI radio map is constructed in the offline phase. In the online-positioning phase, real-time WIF-RSSI is sent to the server to match the radio map and the user’s position is then calculated.

The structure of LSS-RM is shown in Figure 1. The preassigned site-survey trajectory was told to volunteers, and then two smartphones were mounted on the waist (Phone-W) and the foot (Phone-F), respectively. The movement of the volunteer was recorded using a smartphone-embedded MEMS accelerometer, gyroscope, and magnetometer. WiFi-RSSI was also scanned by Phone-W and Phone-F at the same time. Volunteers did not need to take care of their walking frequency or step stride, but just took two smartphones to walk along the preassigned site-survey trajectory, and the WiFi-RSSI radio map was built up automatically. Phone-F could detect zero velocity and the IEZ-PDR algorithm was implemented to calculate the position of volunteers. The angular rate energy-detection algorithm using Phone-W motion data was applied to detect corners of the preassigned site-survey trajectory. Positioning results, stance phase estimation results, and WiFi-RSSI were transferred to the server in real time through the public 4G long term evolution (LTE) network for the post calibration process. In this process, a stance-phase detection-based algorithm is used to count steps and to estimate stride length. With accurate corner detection and preassigned site-survey trajectory information, we could calculate the accurate heading of the volunteers. Finally, with accurate step numbers, stride length, and heading, precise RP coordinates were calculated. The bridge between RP coordinates and WiFi-RSSI in LSS-RM is the start and end timestamp of each stance phase. After the two processes of the offline site-survey phase, a WiFi-RSSI radio map was built up. In the online-positioning phase, when a user sent a demand for the position service, real-time WiFi-RSSI was collected from the user’s smartphone, and a matching algorithm like k-nearest neighbor (KNN) was used to obtain the user’s position. Finally, the real-time localization results were sent back to the user’s smartphone.

**Figure 2:**
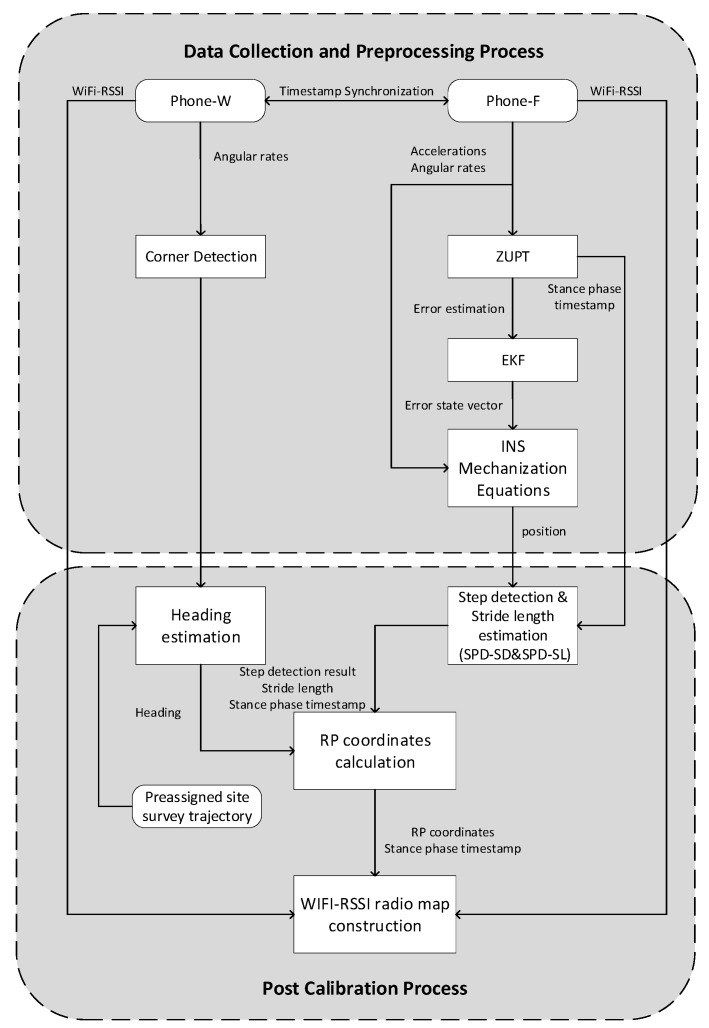
Flowchart of the WiFi-RSSI LSS-RM.

The flowchart of LSS-RM is shown in Figure 2. It has the following nine steps. Steps (1) to (5) happen in the data collection and preprocessing process, and Steps (6) to (9) happen in the post calibration process.

(1) The volunteer is told they should walk along the preassigned site-survey. The server analyzes whether the volunteer walks in the right way.
(2) Two smartphones are mounted on the foot (Phone-F) and waist (Phone-W) of the volunteer, respectively. Motion data of the volunteer, such as accelerometer readings, gyroscope readings, and magnetometer readings are recorded in a format {Timestamp, Triaxial Accelerations, Triaxial Angular Rates, Triaxial Magnetic Field Strength}. Simultaneously, WiFi-RSSI data are recorded by both smartphones in a format {Timestamp, WiFi-RSSI Vector}. The timestamp can be used as a medium to connect the two different kinds of data.
(3) The timestamp difference of Phone-F and Phone-W is measured. Then, a timestamp-synchronization process is taken to align data from the two smartphones.
(4) The position of the volunteer is calculated using the IEZ-INS method based on the accelerometer readings, gyroscope readings, and magnetometer readings of Phone-F. In this step, the stance-phase result of ZUPT is very important and will be used in the post calibration process.
(5) The angular-rate energy detector (ARE) is used to detect the corner based on gyroscope readings of Phone-W. The corner-detection result can be used to calculate the heading with the preassigned site-survey trajectory in the post calibration process.
(6) Step number and stride length are estimated based on stance-phase detection from Phone-F.
(7) Heading of the volunteer is calculated based on preassigned site-survey estimation and corner-detection result from Phone-W.
(8) RP coordinates are calculated using the post calibrated step number, stride length, and heading based on the PDR-INS method.
(9) A radio map is built up with RP coordinates and WiFi-RSSI vectors in a traditional radio map format {RP coordinates, WiFi-RSSI vectors}. The bridge between RP coordinates and WiFi-RSSI vectors in the LSS-RM method is the start and end timestamp of each stance phase.

The offline site-survey phase of WiFi-fingerprinting-based IPS is a trade-off between manpower and radio-map accuracy. Like other methods, our LSS-RM method also aims to find a balance in the trade-off. Comparing with other methods, the advantage of LSS-RM is that no extra devices except for smartphones are needed, and volunteers don't need to take care of their step frequency or stride length to reduce the time-consumption of the offline site survey.

However, LSS-RM still has some drawbacks. Firstly, the system is more complex. Two smartphones are needed in LSS-RM. Despite the advantage of better RP coordinates and denser WiFi-RSSI, the system is more complicated than the traditional one. Secondly, the preassigned site-survey trajectory is needed, and the volunteer must walk along it. Thirdly, considering that the scanning time of LSS-RM on each RP is shorter than in manual site surveys, initial positioning accuracy will be lower. However, with more volunteers joining in the offline phase, positioning accuracy increases. Fourthly, the reliability of LSS-RM depends on the accuracy of RP-coordinate estimation, which can be influenced by many factors like the drift of the MEMS-IMU, the timestamp-alignment accuracy of the two smartphones, and the accuracy of the preassigned site-survey trajectory. With the post calibration process, reliability can be improved. In conclusion, there is still a long way to realize the complete site-survey free WiFi-RSSI radio-map construction method.

## 3. Data Collection and Preprocessing Process

### 3.1. Timestamp Alignment

**Figure 3:**
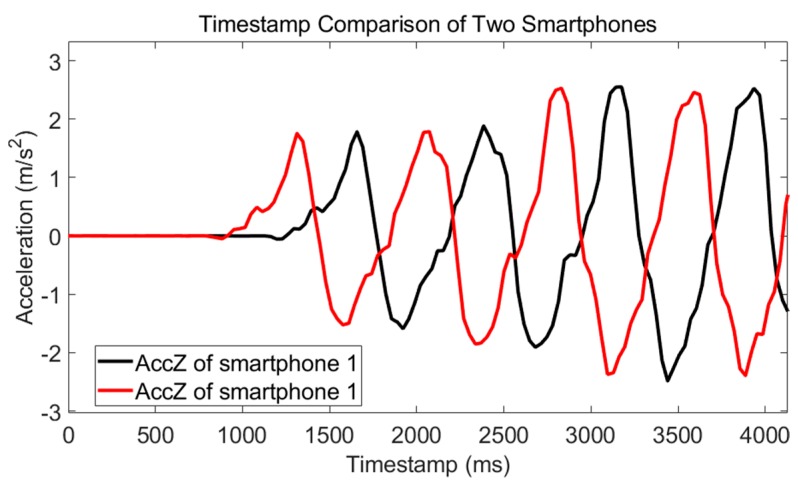
Timestamp comparison of the two smartphones. We tied together two smartphones and shook them along the vertical direction. If their timestamps were synchronous, the timestamp of the first peak of acceleration waveform of *Z*-axis should have been nearly the same, but there existed a timestamp difference Δt and the timestamp should have been synchronized between different smartphones.

Timestamp alignment is the first step of all multidevice-model systems. In our Android APP, timestamps are recorded along with the data. Each timestamp is an index to mark the time when data collected. The timestamp of Android platforms is Unix time that starts from 00:00:00 Coordinated Universal Time (UTC), Thursday, 1 January, 1970. Therefore, in principle, timestamps of different smartphones can be easily aligned because they are under the same time system. However, there are still differences between different smartphones. We performed an experiment, and the result is shown in Figure 3. To simplify *X*-axis of the figures in this paper, the timestamp sequence has been subtracted by the first timestamp. Two smartphones are tied together and move along the vertical direction. If their timestamps are synchronous, the first peak of acceleration waveform of *Z*-axis should has the same timestamp, but there exists a difference, Δt. Therefore, timestamps should be synchronized between different smartphones.

The timestamp-synchronization algorithm is simple in LSS-RM. Firstly, the two-norm of the triaxial accelerations of Phone-F and Phone-W is calculated:

(1) $$Acc_{f}(k)=\sqrt{Acc_{f}^{x}(k)+Acc_{f}^{y}(k)+Acc_{f}^{z}(k)}\;$$

(2) $$Acc_{w}(k)=\sqrt{Acc_{w}^{x}(k)+Acc_{w}^{y}(k)+Acc_{w}^{z}(k)}\;$$

where *k* is the timestamp. $Acc_{f}$ and $Acc_{w}$ are the two-norm of triaxial accelerations of Phone-F and Phone-W, respectively. $Acc_{f}^{x}$, $Acc_{f}^{y}$, $Acc_{f}^{z}$, $Acc_{w}^{x}$, $Acc_{w}^{y}$, $Acc_{w}^{z}$, are *X*-axis, *Y*-axis, and *Z*-axis acceleration of Phone-F and Phone-W, respectively. The timestamp difference is calculated using the difference of the first acceleration peak between Phone-W and Phone-F:

(3) $$\Delta t=timestamp_{f}^{1}-timestamp_{w}^{1}+\varepsilon_{1}+\varepsilon_{2}\;$$

where Δt is the timestamp difference between Phone-W and Phone-F; ${\mathrm{timestamp}}_{f}^{1}$ is the timestamp of the 1st peak of $Acc_{f}$; and ${\mathrm{timestamp}}_{w}^{1}$ is the timestamp of the 1st peak of $Acc_{w}$. $\varepsilon_{1}$ is the timestamp alignment error caused by the sampling process. $\varepsilon_{2}$ is the timestamp alignment error caused by the asynchronous motion of different parts of the body. Finally, timestamps of the two smartphones are aligned with a translation using Δt.

Three tests were performed to test the timestamp-alignment method. The results shown in Table 1 reveal that the Δt of different smartphones is not a constant. It can even reach 1189 ms, which means the positioning result of two smartphones could be over 2 m with a normal walking speed of 2 m/s. The timestamp alignment algorithm of LSS-RM can estimate the timestamp difference of Phone-W and Phone-F.

We want to discuss the influence of $\varepsilon_{1}$ and $\varepsilon_{2}$ in this section. The maximum of $\varepsilon_{1}$ is two times the sampling time. The sampling frequency of MEMS-IMU of our APP was set to 30 Hz, which means the timestamp alignment error was within (–66 ms, 66 ms). The positioning error caused by $\varepsilon_{1}$ was 13.2 cm if the walking speed were 2 m/s. It is quite a small error considering that the positioning accuracy of WiFi-fingerprinting-based IPS is 1–5 m.

**Figure 4:**
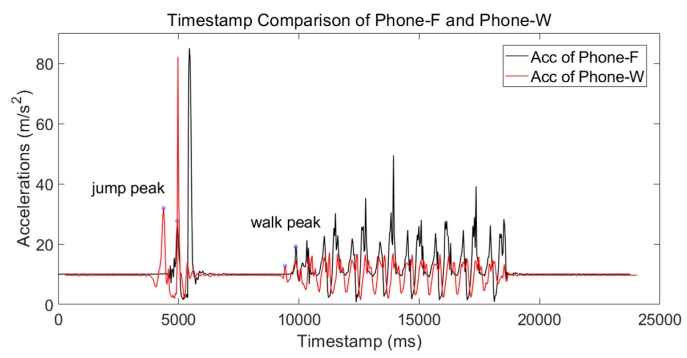
Timestamp comparison of Phone-F and Phone-W. The volunteer jumped at the beginning and stood still for several seconds before walking. The jump peaks are more distinguishable than the walk peaks.

Although Phone-F and Phone-W were mounted on different parts of the body, the motion of the body was almost coordinated. To reduce $\varepsilon_{2}$, the volunteer could have a jump at the beginning of the walk. A test was taken to show a time trace of the two mounted smartphones. The volunteer jumped at the beginning and stood still for several seconds before walking. The result is shown in Figure 4. It is obvious that the jump peaks are more distinguishable than the walk peaks. The timestamp difference calculated using jump peaks was 563 ms (regarded as the reference timestamp error) compared with 473 ms of walk peaks. $\varepsilon_{2}$ was 90 ms for this test. The positioning error caused by $\varepsilon_{2}$ was 18 cm if the walking speed were 2 m/s. Similar with $\varepsilon_{1}$, it is a small error and has little influence on LSS-RM.

### 3.2. Foot-Mounted Inertial Navigation Using Zero-Velocity Update-Aided Extended Kalman Filter (IEZ-INS)

**Figure 5:**
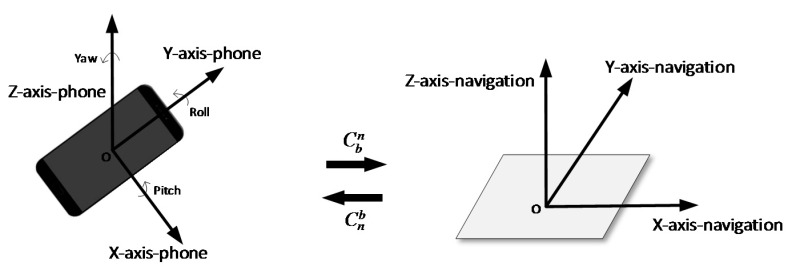
Sketch map of transformation between different coordinate frames. The coordinate frame of the smartphone (b-frame) is fixed, *Y*-axis directs to the head of the phone, *Z*-axis directs up perpendicular to the screen, and the *X*-axis was determined according to the right-hand screw rule. The navigation-coordinate frame (n-frame) used in our method is the east–north–up (ENU) coordinate system. $C_{b}^{n}$ was used to transfer the data from b-frame to n-frame and $C_{n}^{b}$ is from n-frame to b-frame.

The outputs of Phone-F-embedded MEMS-IMU are in the sensor body coordinate frame (b-frame) and should be transferred to the navigation coordinate frame (n-frame) using a rotation matrix $C_{b}^{n}$. The definition of b-frame and n-frame are shown in Figure 5.

The b-frame is determined by the smartphone and usually defined as a right-handed Cartesian coordinate system. *Y-axis*, directs to the head of the phone, *Z-axis*, directs up perpendicular to the screen, and the *X-axis*, was determined according to the right-hand screw rule. Considering the convenience of usage, the n-frame applied in our system is the local east–north–up (ENU) Cartesian coordinate system whose origin is the same as b-frame. The east was labelled *X-axis*, the north *Y-axis,* and the up *Z-axis*. The MEMS-IMU readings, including acceleration, angular rate, and magnetic-field strength are in b-frame and should be transferred to ENU to derive velocity and position. The two different coordinate systems are transferred through the rotation matrix $C_{b}^{n}$. Details of how to use accelerometer and magnetometer readings to calculate the rotation matrix can be found in Reference [17].

After MEMS-IMU readings transferred from the b-frame to the n-frame using $C_{b}^{n}$, the accelerometer, gyroscope, and magnetometer readings of Phone-F could be used in the INS mechanization equations to calculate the volunteer’s position. Firstly, gravity should be subtracted from accelerometer readings in n-frame. Then, the position is calculated with the gravity-free acceleration value. At last, the orientation of the MEMS-IMU is updated with the gyroscope readings. These equations take slightly different forms in different navigation frames. The basic equation utilizing accelerometers and gyroscopes to calculate position is [50]:

(4) $$\left[\begin{array}{c} \begin{array}{c} v_{k}\\ p_{k} \end{array}\\ q_{k} \end{array}\right]=\left[\begin{array}{c} \begin{array}{c} v_{k-1}+(q_{k-1}a_{k}q_{k-1}^{-1}-g)dt_{k}\\ p_{k-1}+v_{k-1}dt_{k} \end{array}\\ \Omega (\omega_{k}dt_{k})q_{k-1} \end{array}\right]\;$$

where *k* is a timestamp, *g* is the gravity, $v_{k}$ is the velocity of the pedestrian, $a_{k}$ is the accelerometer readings, $p_{k}$ is the position of the person, $q_{k}$ is the quaternion describing the orientation frame, dt is the time differential, and Ω(·) is the quaternion update matrix.

Considering the drift of the low-cost smartphone-embedded MEMS-IMU, accumulative error would rise rapidly only by using Equation (4). To solve this problem, the velocity of the stance phase was used as the measurement of the Extended Kalman Filter. This INS with the ZUPT-aided EKF method is called IEZ [16]. The error-state vector of this system is a 15-element vector, δx=[δr,δv,δφ,δa,δω], where δr is the position error, δv is the velocity bias, δφ is the attitude error, δa is the accelerometer bias, and δω is the gyroscope error. In addition, δr,δv,δφ,δa,δω are all three-dimensional vectors. The state-transition matrix *F* is:

(5) $$F=\left[\begin{array}{ccccc} I & I\cdot \Delta t & O & O & O\\ O & I & St\cdot \Delta t & C_{b}^{n}\cdot \Delta t & O\\ O & O & I & O & -C_{b}^{n}\cdot \Delta t\\ O & O & O & I & O\\ O & O & O & O & I \end{array}\right]\;$$

where Δ*t* is the sample interval, and *O* and *I* are the three-dimensional null matrix and unit matrix, respectively. *St*(*k*) is the skew-symmetric matrix of acceleration:

(6) $$St(k)=\left[\begin{array}{ccc} 0 & -a_{z}(k) & a_{y}(k)\\ a_{z}(k) & 0 & -a_{x}(k)\\ -a_{y}(k) & a_{x}(k) & 0 \end{array}\right]\;$$

The measurement model is:

(7) z(k)=Hδx(k)+n(k) 

where z(k) is the measurement, δx is the error-state vector at timestamp *k*, n(k) is the measurement-noise vector at the timestamp *k*, and *H* is the measurement matrix:

(8) $$H=\left[\begin{array}{ccccc} O & I & O & O & O \end{array}\right]\;$$

Steps of IEZ can be found in References [16,17]. An experiment was conducted to validate the IEZ-INS algorithm. A volunteer walked along a rectangular corridor and walked back to the start point. The experimental setup is summarised in Table 2. The MI6 smartphone was mounted on the left foot, and the sampling frequency was 30 Hz.

**Figure 6:**
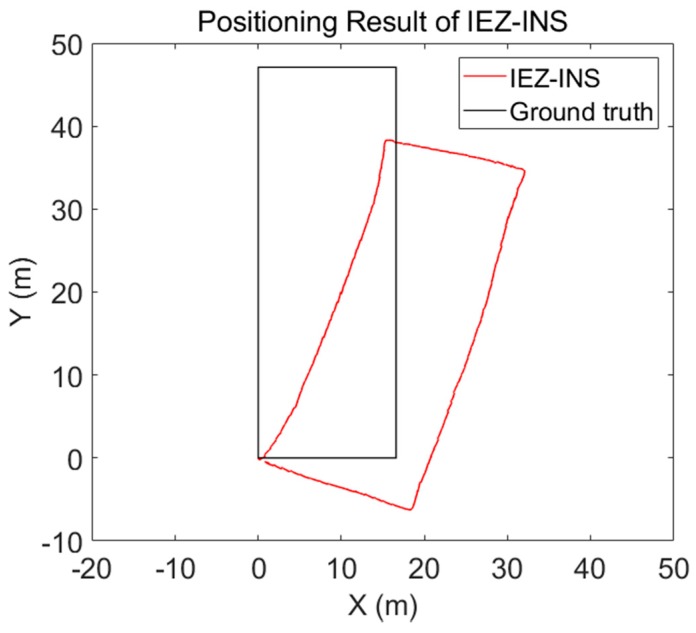
Positioning result of Foot-Mounted Inertial Navigation Using Zero-Velocity Update-Aided Extended Kalman Filter (IEZ-INS).

The positioning result is shown in Figure 6. It is obvious that the positioning result is badly influenced by the heading error. Therefore, the post calibration process was needed to get accurate RP coordinates.

### 3.3. Stance-Phase Detection Using Phone-F-Embedded MEMS-IMU

The velocity error estimated during the stance phase is the measurement vector in IEZ. Considering that positioning errors will accumulate fast due to sensor drift, zero-velocity information is efficient in error correction. Furthermore, in our LSS-RM method, the stance phase is used to count steps and estimate stride length in the post calibration process.

To get more robust stance detection, three ZUPT detectors were fused; stance phase occurs when the results of all three detectors were in the stance phase. These three detectors used in this paper are from Reference [13]: the acceleration moving-variance detector (MV), the acceleration magnitude detector (MAG), and the ARE.

(9) $$T_{mv}(k)=\frac{1}{W}\sum_{n=k}^{k+W-1}\frac{1}{\sigma_{a}^{2}}{\left\|a(n)-\bar{a(k)}\right\|}^{2}\;$$

(10) $$T_{mag}(k)=\frac{1}{W}\sum_{n=k}^{k+W-1}\frac{1}{\sigma_{a}^{2}}{\left(\left\|a(n)\right\|-g\right)}^{2}\;$$

(11) $$T_{are}(k)=\frac{1}{W}\sum_{n=k}^{k+W-1}\frac{1}{\sigma_{\omega }^{2}}{\left\|\omega (n)\right\|}^{2}\;$$

where *k* is a time index. W is the window length. g is gravity. $\sigma_{a}^{2}$ and $\sigma_{w}^{2}$ denote the accelerometer and gyroscope noise variance. ‖·‖ is the 2-norm calculation. $T_{mv}$, $T_{mag}$,$\;\mathrm{and}\;T_{are}$ are the test statics of the MV detector, MAG detector, and ARE detector, respectively. $\bar{a\left(k\right)}$ is the average of *a* during the average window *W* at time index *k*:

(12) $$\bar{a(k)}=\frac{1}{W}\sum_{n=k}^{k+W-1}a(n)\;$$

**Figure 7:**
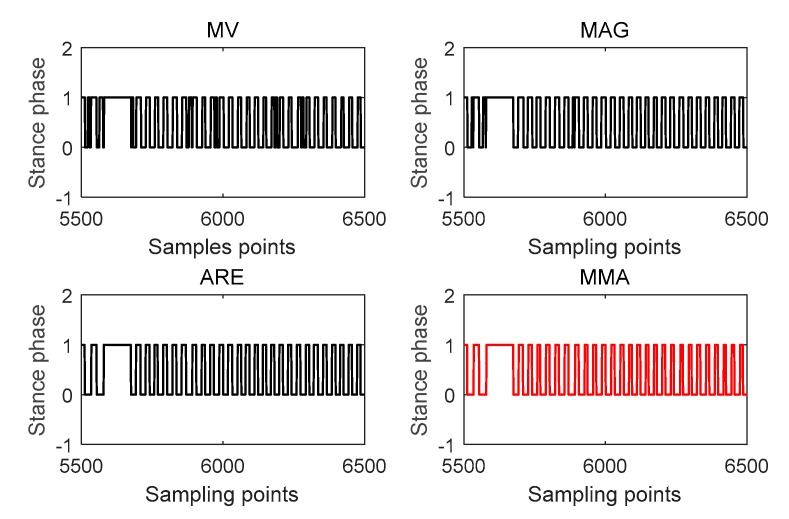
Stance-phase estimation results of acceleration moving-variance (MV), acceleration magnitude (MAG), angular-rate energy (ARE), and MMA detectors. 1 and 0 represent the pedestrian is in the stance phase and swing phase, respectively.

$T_{mv}$ and $T_{mag}$ use accelerometer readings, while $T_{are}$ uses gyroscope readings. The stance phase occurs when all these three ZUPT detectors are below their thresholds:

(13) $$\begin{array}{l} MV(k)=\{\begin{array}{lll} 1 & if & T_{mv}(k)<\gamma_{mv}\\ 0 & if & T_{mv}(k)\geq \gamma_{mv} \end{array}\\ MAG(k)=\{\begin{array}{lll} 1 & if & T_{mag}(k)<\gamma_{mag}\\ 0 & if & T_{mag}(k)\geq \gamma_{mag} \end{array}\\ ARE(k)=\{\begin{array}{lll} 1 & if & T_{are}(k)<\gamma_{are}\\ 0 & if & T_{are}(k)\geq \gamma_{are} \end{array}\\ MMA(k)=MV(k)\&MAG(k)\&ARE(k) \end{array}$$

where MV(k), MAG(k), and ARE(k) are stance-phase estimation results. $\gamma_{mv}$, $\gamma_{mag}$, and $\gamma_{are}$ are the threshold of MV, MAG, and ARE, respectively. MMA(k) is the combination of the previous three detectors and MMA is short for the first letters of MV, MAG, and ARE. An experiment was taken to verify the stance phase estimation result of different detectors. The results shown in Figure 7 depict that, in this experiment, stance-phase detectors using accelerometer readings have some errors and ARE using gyroscope readings perform better. This is not always right and in some other scenarios, like taking an elevator, MV and MAG may have a better performance. In any case, MMA will always have the best stance-phase estimation among MV, MAG, and ARE.

### 3.4. Corner Detection Using Phone-W-Embedded MEMS-IMU

The gyroscope readings of the waist-mounted smartphone can be used to detect the timestamp when a volunteer walks around a corner. The ARE method shown in Equation (11) is used:

(14) $$Corner(k)=\{\begin{array}{lll} Corner(k-1)+1 & if & \frac{1}{W}\sum_{n=k}^{k+W-1}\frac{1}{\sigma_{\omega }^{2}}{\left\|\omega (n)\right\|}^{2}>\gamma_{corner}\\ Corner(k) & if & \frac{1}{W}\sum_{n=k}^{k+W-1}\frac{1}{\sigma_{\omega }^{2}}{\left\|\omega (n)\right\|}^{2}\leq \gamma_{corner} \end{array}\;$$

where Corner(k) is the corner-detection result, and $\gamma_{corner}$ is the threshold of corner detection. Considering that the turning speed of each site-survey process is different, the threshold $\gamma_{corner}$ is not a fixed value. It is chosen as 10 times the average value of the whole ARE detector sequence:

(15) $$\gamma_{corner}=10\times \frac{\sum_{k=1}^{N}T_{are}(k)}{N}\;$$

where $T_{are}$ is the ARE detector of Equation (11), and N is the length of $T_{are}$.

**Figure 8:**
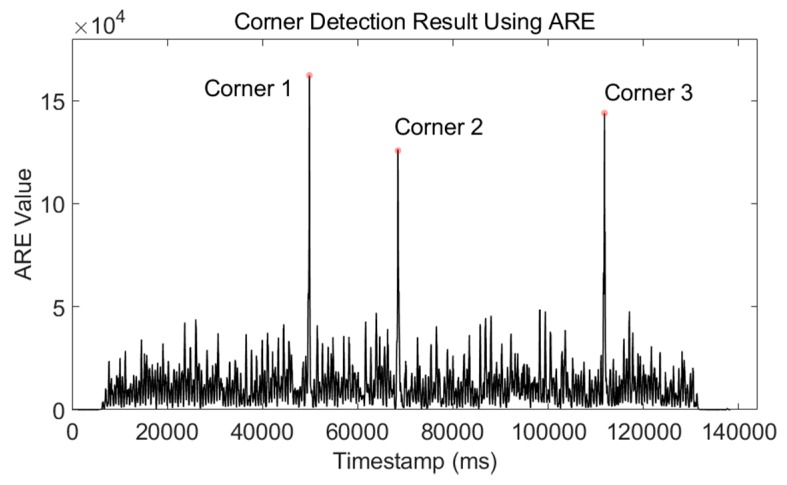
Corner-detection result using the ARE detector. This experiment was performed with the user walking around a square corridor and doing three turns. From the ARE values, we can clearly pick out the corners.

The ARE detector of Phone-W can clearly distinguish the difference of walking a straight line and tuning around a corner, which is shown in Figure 8.

Three more experiments were conducted to verify the corner-detection algorithm. The volunteer walked around a square corridor for one, three, and six turns, respectively. The true and estimated number of corners of these three tests is summed in Table 3. The results show that the ARE-based corner-detection algorithm can accurately estimate turning movement and provide the timestamp when a turning movement occurs. We took a smartphone to record the video of the volunteer walking along the preassigned site-survey trajectory. From the video, we can take the average timestamp of the turning motion as the reference timestamp. All timestamp differences were smaller than 500 ms, which is smaller than the time duration of one step and has little influence on RP-coordinate estimation.

**Figure 9:**
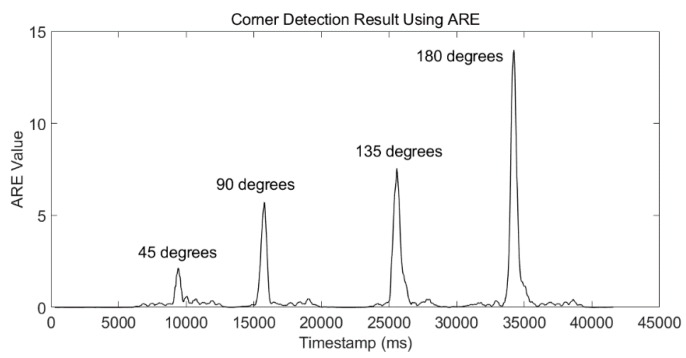
Corner-detection result using an ARE detector. The volunteer turned 45 degrees, 90 degrees, 135 degrees, and 180 degrees during a walk. The four corners were detected correctly.

This corner-detection method is not only applicable to 90-degree corners. A test was conducted to validate our method in other situations. The volunteer turned 45 degrees, 90 degrees, 135 degrees, and 180 degrees during a walk. The result is shown in Figure 9. The four typical degrees of corners can be detected correctly. We need to point out that, if the degree of the corner is too small, this corner-detection algorithm may have a wrong estimation. However, we can avoid this situation with a proper preassigned site-survey trajectory design.

## 4. Post Calibration Process

### 4.1. Stance-Phase Detection Based on Step Detection (SPD-SD)

**Figure 10:**
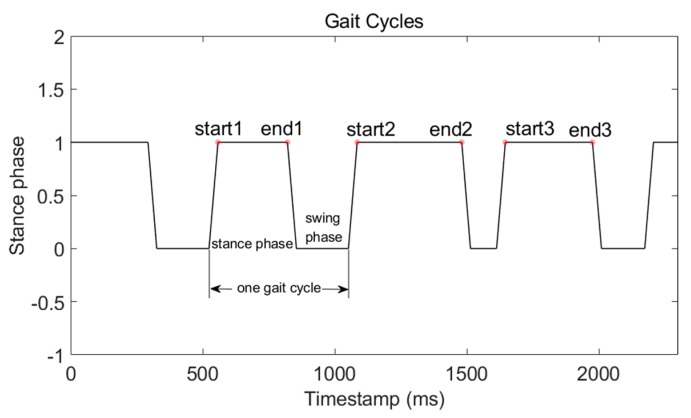
Five complete gait cycles during a walk. Each gait cycle consists of a stance phase and a swing phase. The start timestamp and the end timestamp of the stance phase can be detected from the rising edge and falling edge, respectively.

One gait cycle consists of a stance phase and a swing phase. Five complete gait cycles are shown in Figure 10. It is very intuitive that we can count steps through counting stance phases. This method is SPD-SD. Furthermore, the start and end timestamp of the stance phase can be used to withdraw WiFi-RSSI from the complete WiFi-RSSI sequence and mark it with RP coordinates.

**Figure 11:**
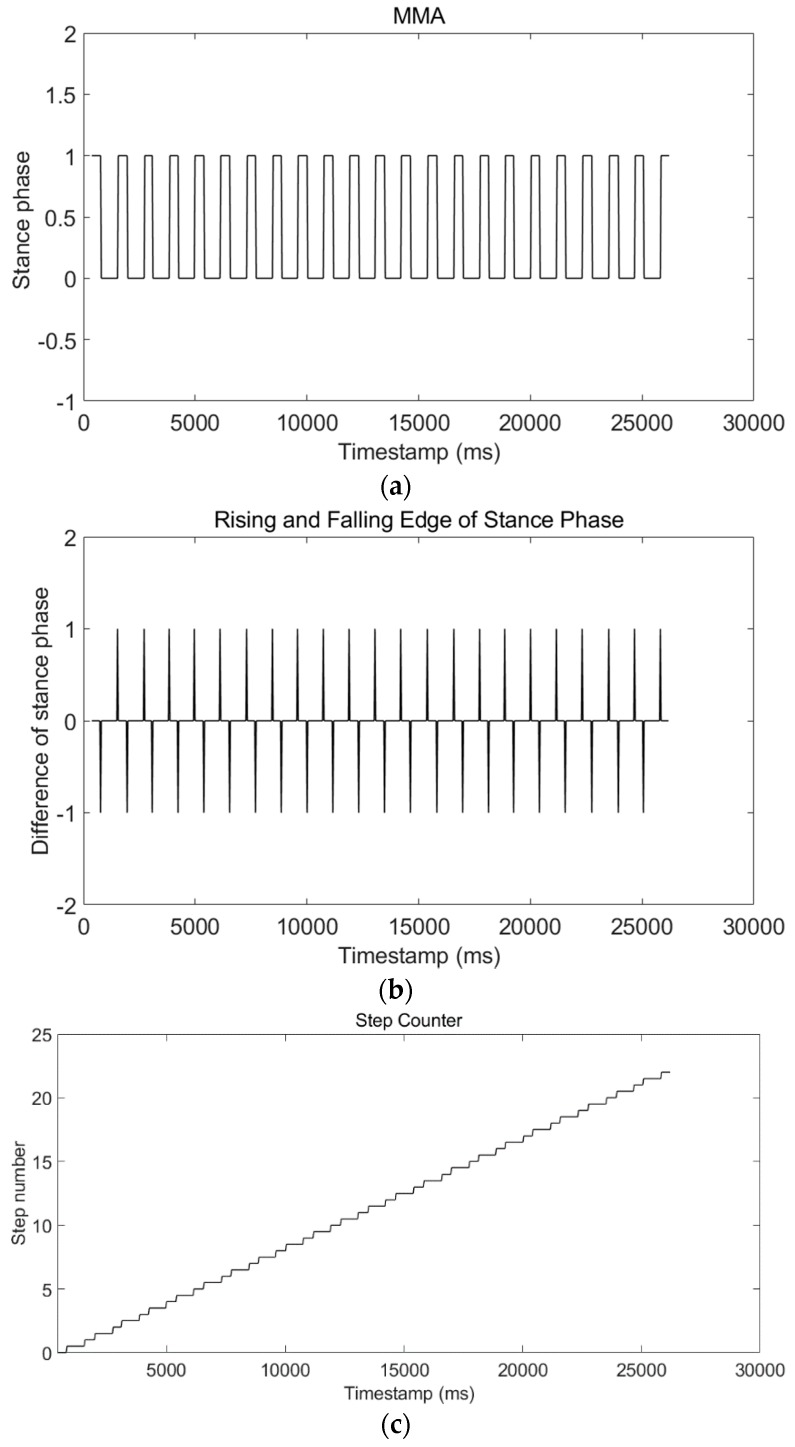
Results of the stance-phase detection based on step detection (SPD-SD) algorithm applied to a test of walking 22 steps. (**a**) Waveform of stance phase using the MMA algorithm. (**b**) Rising and falling edge of stance-phase waveform. 1 is the rising edge and −1 is the falling edge. (**c**) Step-detection result using Equation (16) and final step detection was 22 steps, which was the same as the true number.

The equation of the SPD-SD method is:

(16) $$step(k)=\{\begin{array}{ccc} step(k-1)+0.5 & if & ARE(k)-ARE(k-1)=1\\ step(k-1)+0.5 & if & ARE(k)-ARE(k-1)=-1\\ step(k-1) & if & ARE(k)-ARE(k-1)=0 \end{array}\;$$

where step(k) is the step-detection result at timestamp *k*. The volunteer’s movement always starts from the stance phase and ends with the stance phase, which means starting from a falling edge and ending with a rising edge of the stance-phase waveform. Therefore, the result of SPD-SD, which is equal to step(end), must be an integer. We took a test walking 22 steps. Figure 11a shows the stance-phase waveform. Figure 11b shows the rising and falling edge of the stance-phase waveform. Figure 11c shows the step-detection result using Equation (16). The step-detection result is 22 steps, which is the same as the true number.

To better verify the SPD-SD algorithm, we performed a much longer test, and the length of the trajectory was around 500 m. This test was repeated three times. A volunteer took a camera and recorded a video to count the true step number. The results are shown in Table 4. The step-detection results of the first two tests were the same as the true number. However, the step error of the third test was 88. The reason is that in the third test the movement mode of the volunteer was running, not walking. The stance-phase detector with a fixed threshold had limited performance. In Reference [17], we developed a MAG-ZUPT method to estimate the stance phase of running using magnetic-field strength. In Reference [51], we developed an adaptive-threshold method of walking and running stance-phase detection. These two methods can solve the stance-phase detection problem but add extra sensors or increase computation complexity. Luckily, unlike the first responders, site-survey volunteers did not need to run, and the SPD-SD algorithm could provide good performance.

### 4.2. Stance-Phase Detection-Based Stride-Length Estimation (SPD-SL)

The positioning result is the moving trail of Phone-F, which touches the ground only in the stance phase. Therefore, we can calculate the position in stance phase, and the distance between the neighbor stance-phase positions is stride length. This is the fundamental principle of the SPD-SL algorithm. To reduce fluctuation during stance phase, the position of each stance phase was averaged, with the window length calculated with the start and end timestamp of the stance phase:

(17) $$spwl(k)=sp_{k}(end)-sp_{k}(start)\;$$

where spwl(k) is the number of timestamps of the *k-*th stance phase, $sp_{k}\left(end\right)$ is the end timestamp of the *k-*th stance phase, and $sp_{k}\left(start\right)$ is the start timestamp of the *k-*th stance phase. The coordinates of the *k-*th stance phase are averaged with spwl(k):

(18) $$spc(k)=\frac{1}{spwl(k)}\times \sum_{i=sp_{k}(start)}^{sp_{k}(end)}traj(i)\;$$

where *spc*(*k*) are the average coordinates of the *k-*th stance phase, and *traj* are the coordinates of the whole trajectory. The stride length between the (*k* − 1)*-*th stance phase and the *k-*th stance phase are calculated using:

(19) $$sl(k)=\sqrt{{\left(spc_{x}(k)-spc_{x}(k-1)\right)}^{2}+{\left(spc_{y}(k)-spc_{y}(k-1)\right)}^{2}}\;$$

where *sl*(*k*) is the *k-*th step length, *spc_x_* and *spc_y_* are the *x* and *y* values of *spc*, respectively.

**Figure 12:**
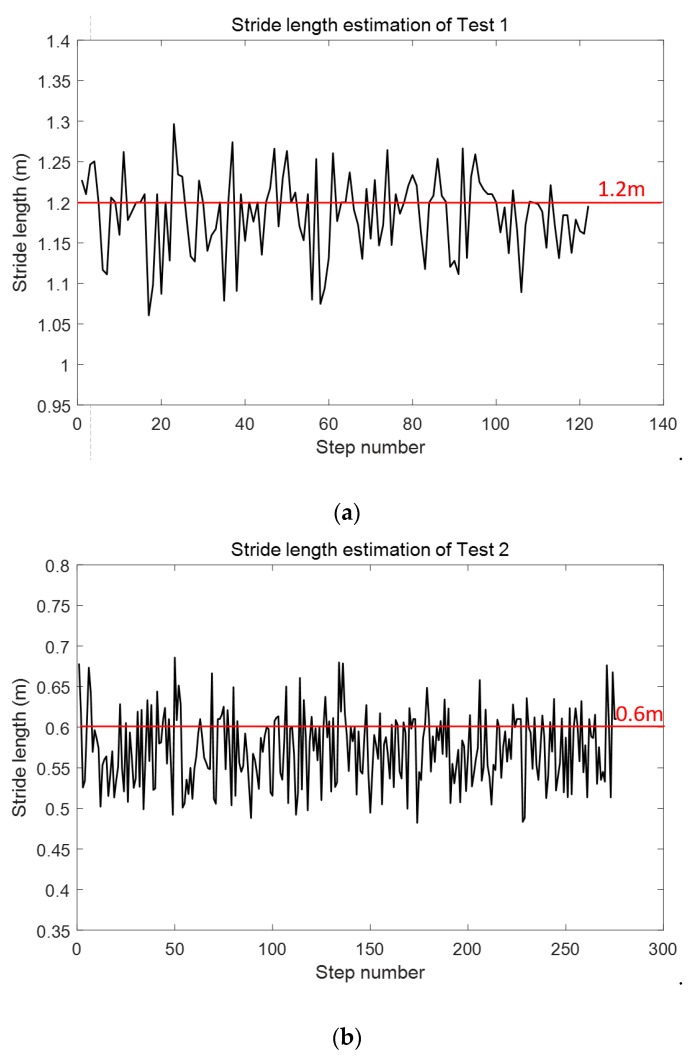
Stride-length estimation results of Test 1 and Test 2. (**a**) Result of Test 1. The volunteer walked 122 steps trying to keep stride length to 1.2 m. (**b**) Result of Test 2. The volunteer walked 276 steps trying to keep stride length to 0.6 m.

Considering that the smartphone was mounted on one foot, the stride length of SPD-SL was the stride length between the same foot, which was nearly two times longer than the stride length between the left and right foot. We took two tests to verify the SPD-SL algorithm. Test had a stride length of 1.2 m, and Test 2 of 0.6 m. The stride length of each step is shown in Figure 12. Comparison of true stride length and estimated stride length is shown in Table 5. The stride length estimation errors of Test 1 and Test 2 are 0.02 m and 0.03 m, respectively. It must be pointed out that it is difficult to have an accurate measurement of true stride length of each step. The volunteer tried the best to keep a constant stride length. However, considering that the positioning error of WiFi-fingerprinting based IPS is usually larger than 1 m, the stride length estimation accuracy using SPD-SL is enough to calculate RP coordinates.

### 4.3. Post Calibration with Preassigned Site-Survey Trajectory

Pedestrian dead-reckoning-based inertial navigation system (PDR-INS) integrates step lengths and heading estimations at each detected step to compute the position of the pedestrian [18]. The relationship between the *k-*th step and the (*k* − 1)*-*th step is:

(20) $$\{\begin{array}{c} x(k)=x(k-1)+l(k)*\cos(\varphi (k))\\ y(k)=y(k-1)+l(k)*\sin(\varphi (k)) \end{array}\;$$

where *k* is the step number calculated using the SPD-SD algorithm and can be mapped to the timestamp. [*x*(*k*), *y*(*k*)] and [*x*(*k* − 1), *y*(*k* − 1)] are position coordinates of *k-*th step and (*k* − 1)*-*th step, respectively. *l* is the stride length calculated using the SPD-SL algorithm. φ is the heading calculated using corner-detection results and the preassigned site-survey trajectory. Our heading-estimation algorithm is very intuitive. With the knowledge that the volunteer cannot cross the wall, when they meet the corner they have to turn in the direction of the corridor. Therefore, the corner detected by Phone-W using gyroscope reading is an indicator of heading change and the value of the heading difference can be easily obtained using the preassigned site-survey trajectory.

Finally, with accurate RP coordinates and the timestamp duration of each stance phase, the WiFi-RSSI radio map is constructed. The WIFI-RSSI radio map can be built up with the correct RP coordinates and the corresponding timestamp. The time interval, shown in Equation (17), between the start and the end timestamp of each stance phase is the bridge to connect RP coordinates and WiFi-RSSI vectors.

**Figure 13:**
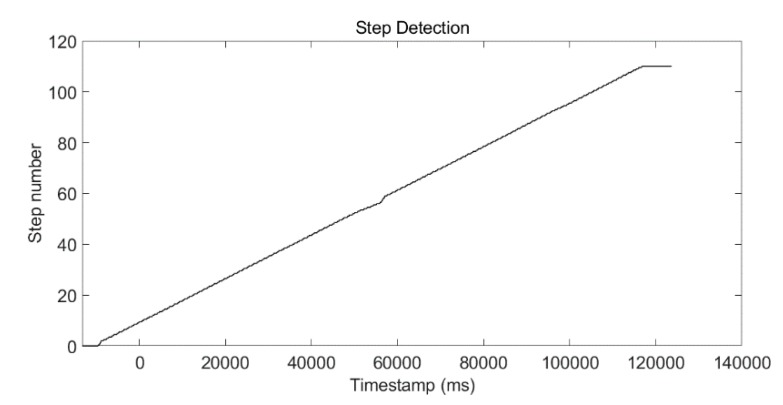
Step detection using the SPD-SD algorithm.

An experiment was conducted to show the post calibration process. The test site was a square corridor. The step-detection result using the SPD-SD algorithm is shown in Figure 13, and the total step number was 108, which matched the true step number.

**Figure 14:**
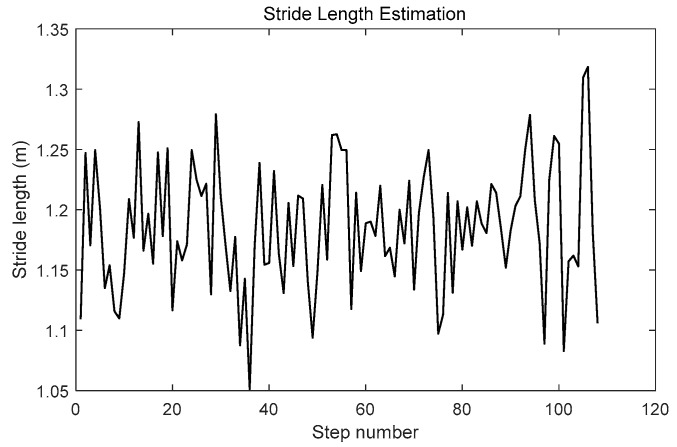
Stride length estimation using the stance-phase detection-based stride-length estimation (SPD-SL) algorithm.

Stride length is estimated using SPD-SL, and the result of each step is shown in Figure 14. The estimated stride length of each step is around 1.2 m, which was the nearly the same as the true stride length.

**Figure 15:**
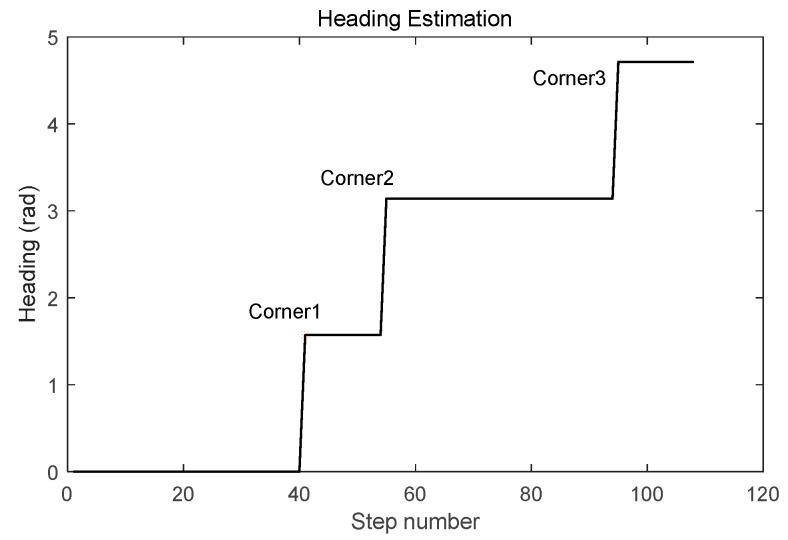
Heading estimation using corner-detection result from Phone-W and preassigned site-survey trajectory.

The heading was estimated using the corner-detection result from Phone-W and the preassigned site-survey trajectory, which is shown in Figure 15. According to the direction of the corridor, when a corner occurs the heading is plus or minus 90 degrees. RP coordinates calculated with the LSS-RM method, positioning results using Phone-F based on IEZ-INS, and the ground truth were compared. The positioning result of Phone-F based on IEZ-INS was badly influenced by the drift of the low-cost MEMS-IMU. However, a series of algorithms, such as SPD-DS, SPD-LS, and corner detection, can calibrate inaccurate IEZ-INS results into accurate RP coordinates.

**Figure 16:**
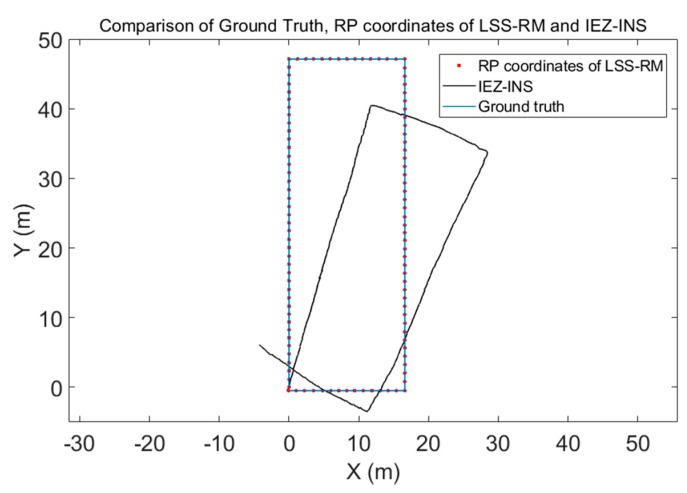
RP coordinates calculated with the LSS-RM method, positioning results using Phone-F based on IEZ-INS, and the ground truth are compared, although the positioning accuracy of the smartphone-embedded low-cost MEMS-IMU is limited. We can still use a series of algorithms in LSS-RM to get accurate RP coordinates.

RP coordinates were calculated using Equation (20), and results are shown in Figure 16, even though the positioning accuracy of the smartphone-embedded low-cost MEMS-IMU is limited. We can still use a series of algorithms in LSS-RM to get the accurate RP coordinates that are a good match with the ground truth.

## 5. Experimental Results

### 5.1. Summary of Submodule Tests in Previous Sections

Firstly, the experimental results of each submodule of our LSS-RM method have already been depicted in the corresponding sections. We would like to have a summary here:

(1) Timestamp alignment: Table 1 shows that our timestamp-alignment algorithm is very efficient. Figure 4 shows the timestamp comparison of Phone-F and Phone-W.
(2) IEZ-INS: Figure 6 shows that the positioning result of IEZ-INS using Phone-F is influenced by the heading error. The post calibration process is needed for accurate RP coordinates.
(3) Corner detection: Figure 8 and Figure 9, and Table 3 show that we can detect corners correctly using ARE.
(4) SPD-SD: Figure 11 and Table 4 show that the SPD-SD algorithm can have accurate step detection.
(5) SPD-SL: Figure 12 and Table 5 show that the SPD-SL algorithm is accurate enough for calculating RP coordinates for WiFi-fingerprinting-based IPS.
(6) Post calibration: Figure 16 is the post calibration result. RP coordinates are matched with the ground truth.

Then, we conducted a comprehensive experiment to compare the WIFI-RSSI radio map built using our LSS-RSS with the one built with the traditional manual site survey method.

### 5.2. Comprehensive Experiment to Verify LSS-RM Method

**Figure 17:**
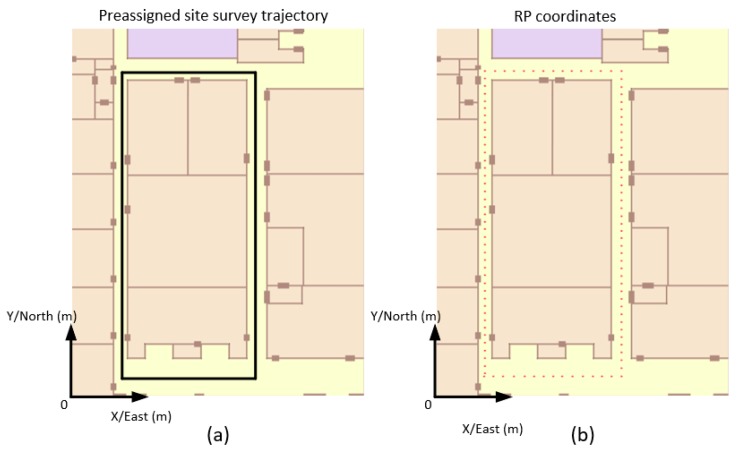
Preassigned site-survey trajectory and RP coordinates. (**a**) Preassigned site-survey trajectory along a square corridor. The volunteer was asked to walking along this trajectory. (**b**) Calculated RP coordinates using timestamp alignment, SPD-SD, SPD-SL, and heading estimation based on corner detection. There are 108 RPs.

This experiment was conducted in F6, New Main Building, Beihang University. The site is a square corridor, and the total length of the corridor is 128 m, shown in Figure 17a. Phone-F and Phone-W that were used in this experiment were MI6 (Xiaomi, Beijing, China), which contains a triaxial accelerometer, a triaxial gyroscope, and a triaxial magnetometer. The operating system of the two smartphones is MIUI based on Android 8.0, and we have developed an Android APP to sample accelerometer readings, gyroscope readings, magnetometer readings, and WiFi-RSSI. The sampling frequency of WiFi-RSSI and MEMS-IMU was 10 Hz and 30 Hz, respectively.

Firstly, as a comparison, the manual site survey was conducted with 108 RPs. The distance between adjacent RP was 1.2 m and the sampling time on each RP was 0.5 min. A manual WiFi-RSSI radio map was built in 54 min. Then a volunteer mounted with Phone-W and Phone-F walked along the preassigned site survey trajectory. RP coordinates, shown in Figure 17b, were calculated using a series of algorithms in LSS-RM like timestamp alignment, SPD-SD, SPD-SL, and heading estimation based on corner detection.

**Figure 18:**
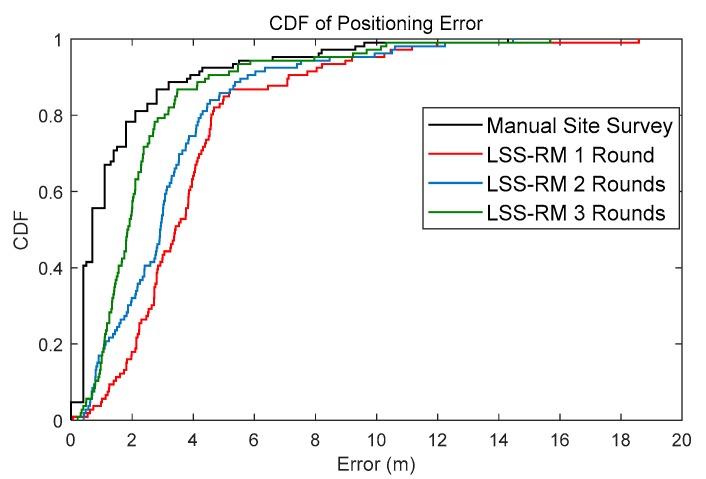
Comparison of CDFs of different radio maps. The black line is the WiFi positioning result using the manual radio map. The red line is the positioning result using our LSS-RM based radio map for one round along the preassigned site-survey trajectory. The blue line is using the LSS-RM-based radio map for two rounds, and green line for three rounds. Although the positioning accuracy of the manual site survey method is higher, its time consumption is nonnegligible. Furthermore, with more volunteers walking more times, positioning accuracy would rise remarkably.

The radio map constructed by LSS-RM was compared with the traditional manual one. The number of test points was also 106 for both the manual radio map and the LSS-RM based radio map. The cumulative distribution function (CDF) plots of the WiFi-fingerprinting-based positioning using the manual radio map and the LSS-RM-based radio map are shown in Figure 18.

The time consumption and average-positioning error of different site-survey methods are summarized in Table 6. The results show that the manual site survey method has the best positioning accuracy, but time consumption is 54 min, which is several times longer than of the LSS-RM method. As for LSS-RM, positioning accuracy rises with more walking, which means more WiFi-RSSI can make the radio map more robust.

The most important advantage of LSS-RM is that it can conspicuously reduce the time consumption of offline site surveys. This characteristic helps the large-scale commercial deployment of WIFI-RSSI indoor positioning systems. Taking the shopping market application as an example, LSS-RM helps to build the WiFi-RSSI radio map in a much shorter time compared with the traditional manual site-survey method. Then, customers can retrieve their positions by matching the WiFi-RSSI collected in real time with the WiFi-RSSI radio map built using LSS-RM. With the position information and the indoor map of the shopping mall, customers can navigate to the nearest shoe shop or exit. Furthermore, shopping-mall managers can use the position information of customers to optimize the arrangement of the stores.

## 6. Conclusions

LSS-RM is developed in this paper to reduce the time consumption of offline site-survey processes. The offline phase of LSS-RM consists of data collection and preprocessing, and post calibration. The use of MEMS accelerometer and gyroscope readings of Phone-F can easily detect the stance phase of the volunteer. Furthermore, stance-phase information can be used to count steps (SPD-SD) and estimate stride length (SPD-SL). Using MEMS gyroscope readings of Phone-W can detect the corner of preassigned site-survey trajectories and accurate headings can be estimated in the post calibration process. The pedestrian dead-reckoning algorithm is used to calculate RP coordinates. A radio map is built with the RP coordinates and WiFi-RSSI vectors in a traditional radio-map format {RP coordinates, WiFi-RSSI vectors}. The bridge between RP coordinates and WiFi-RSSI vectors in the LSS-RM method is the start and end timestamp of each stance phase.

Several experiments were conducted to evaluate the submodules of the LSS-RM method. The results show that timestamp alignment, corner detection using ARE, step detection using SPD-SD, stride estimation using SPD-SL, heading estimation using corner information, preassigned site-survey trajectory, and RP-coordinate calculation all performed well. Finally, a comprehensive experiment was conducted to compare the performance of the traditional manual site survey and the LSS-RM method. The result shows that the manual radio map has better positioning accuracy, while time consumption is 54 min compared with the 2.6–7.8 min of the LSS-RM method. Furthermore, positioning accuracy of the LSS-RM method can be improved by more volunteers joining in the site-survey work. In our future work, we will research a site-survey-free method to construct the WIFI-RSSI radio map.

## Figures and Tables

**Table 1:** Tests of timestamp difference of two smartphones.

Test Number	Timestamp of First Peak of Smartphone 1 (ms)	Timestamp of First Peak of Smartphone 2 (ms)	Timestamp Difference (ms)
1	1532703421657	1532703421312	345
2	1532705867200	1532705866011	1189
3	1532706440599	1532706439882	717

**Table 2:** Experimental setup.

Setup Content	Description
Experiment site	A rectangular corridor
Total length of the corridor	128 m
Mounting place of the smartphone	Left foot
Smartphone used	MI6 from Xiaomi
Sensors used	Triaxial accelerometer, gyroscope, and magnetometer
Sampling frequency	30 Hz

**Table 3:** Tests of ARE-based corner-detection algorithm using a waist-mounted smartphone.

Test Number	True Number of Corners	Estimated Number of Corners	Corner-Detection Error	Average Timestamp Error (ms)
1	3	3	0	324
2	11	11	0	426
3	23	23	0	233

**Table 4:** Tests of SPD-SD algorithm using foot-mounted smartphone.

Test Number	True Number of Steps	Estimated Number of Steps	Error
1	426	426	0
2	437	437	0
3	413	325	88

**Table 5:** Comparison of true stride length and estimated stride length.

Test Number	True Stride Length (m)	Average Estimated Stride Length (m)	Error (m)
1	1.2	1.18	0.02
2	0.6	0.57	0.03

**Table 6:** Comparison of time consumption and average positioning error.

Test Type	Time-Consumption (Minute)	Average Positioning Error (m)
LSS-RM of one round	2.6	3.91
LSS-RM of two rounds	5.1	3.25
LSS-RM of three rounds	7.8	2.47
Manual site survey	54	1.61
